# Cu(II) enhances the effect of Alzheimer’s amyloid-β peptide on microglial activation

**DOI:** 10.1186/s12974-015-0343-3

**Published:** 2015-06-24

**Authors:** Fengxiang Yu, Ping Gong, Zhuqin Hu, Yu Qiu, Yongyao Cui, Xiaoling Gao, Hongzhuan Chen, Juan Li

**Affiliations:** Department of Pharmacology, Shanghai Jiao Tong University School of Medicine, 280 South Chongqing Road, Shanghai, 200025 China; Shanghai Institute of Immunology, Shanghai Jiao Tong University School of Medicine, 280 South Chongqing Road, Shanghai, 200025 China

**Keywords:** Cu(II)-Aβ complex, Aggregation, Microglia, Reactive oxygen species, Tumor necrosis factor-α, Nitric oxide, NF-кB

## Abstract

**Background:**

Aggregated forms of amyloid-β (Aβ) peptides are important triggers for microglial activation, which is an important pathological component in the brains of Alzheimer’s patients. Cu(II) ions are reported to be coordinated to monomeric Aβ, drive Aβ aggregation, and potentiate Aβ neurotoxicity. Here we investigated whether Cu(II) binding modulates the effect of Aβ on microglial activation and the subsequent neurotoxicity.

**Methods:**

Aβ peptides were incubated with Cu(II) at an equimolar ratio to obtain the Cu(II)-Aβ complex. Primary and BV-2 microglial cells were treated with Cu(II)-Aβ, Aβ, or Cu(II). The tumor necrosis factor-α (TNF-α) and nitric oxide levels in the media were determined. Extracellular hydrogen peroxide was quantified by a fluorometric assay with Amplex Red. Mitochondrial superoxide was detected by MitoSOX oxidation.

**Results:**

Incubation of Cu(II) with Aβ confers different chemical properties on the resulting complex. At the subneurotoxic concentrations, Cu(II)-Aβ (but not Aβ or Cu(II) alone) treatment induced an activating morphological phenotype of microglia and induced the microglial release of TNF-α and nitric oxide as well as microglia-mediated neuronal damage. Cu(II)-Aβ-triggered microglial activation was blocked by nuclear factor (NF)-κB inhibitors and was accompanied with NF-κB activation. Moreover, Cu(II)-Aβ induced hydrogen peroxide release, which was not affected by NADPH oxidase inhibitors. Mitochondrial superoxide production was increased after Cu(II)-Aβ stimulation. N-acetyl-cysteine, a scavenger of reactive oxygen species (ROS), inhibited Cu(II)-Aβ-elicited microglial release of TNF-α and nitric oxide as well as the microglia-mediated neurotoxic effect.

**Conclusion:**

Our observations suggest that Cu(II) enhances the effect of Aβ on microglial activation and the subsequent neurotoxicity. The Cu(II)-Aβ-triggered microglial activation involves NF-κB activation and mitochondrial ROS production.

**Electronic supplementary material:**

The online version of this article (doi:10.1186/s12974-015-0343-3) contains supplementary material, which is available to authorized users.

## Background

Neuroinflammation, characterized by excessive glial activation and overproduction of proinflammatory cytokine and chemokines, plays a critical role in the pathogenesis of neurodegeneration in Alzheimer’s disease (AD) and related neurodegenerative disorders [1]. Inhibition of glial activation is shown to improve synaptic dysfunction and behavioral deficits in AD animal models [2].

Amyloid-β (Aβ) peptides are key molecules in AD pathology as they aggregate to form amyloid plaques, the primary neuropathological hallmarks of AD [3]. Aggregated Aβ peptides, in the forms of fibrils and oligomers, are prominent triggers for microglial activation [4–6]. Accumulating evidence demonstrates that the redox-active Cu(II) ions can be coordinated to the histidine (His6, His13, and His14) or Tyr10 of monomeric Aβ. This coordination leads to formation of the Cu(II)-Aβ complex (predominantly with 1:1 stoichiometry), an aggregation process that involves a conformational change [7–9]. Copper homeostasis is profoundly affected in AD. The concentration of extracellular Cu(II), especially within the amyloid plaques, is markedly elevated in AD brains [10, 11]. An elevated Cu(II) concentration in the cerebrospinal fluid from patients with AD has also been reported [12]. Micromolar concentrations of Cu(II) induce dramatic Aβ aggregation at a mildly acidic environment (pH 6.6), a condition that mimics the cerebral acidosis in AD [13]. Therefore, it is possible that excess extracellular Cu(II), via driving Aβ aggregation, participates in AD pathology. The interactions between Cu(II) and monomeric Aβ are suggested to lead to enhancement of Aβ neurotoxicity [14, 15]. It is unknown whether these interactions modulate the effect of Aβ on microglial activation.

The transcription factor nuclear factor (NF)-κB has been identified as a key regulator of cellular immune responses, including microglial activation [16]. NF-κB activation can be directly driven by reactive oxygen species (ROS) [17, 18]. ROS, derived from NADPH oxidase (NOX) or mitochondria, play important roles in regulating microglial responses to various stimuli. ROS from NOX may be involved in microglial activation stimulated by fibrillar Aβ, lipopolysaccharide (LPS), or Zn(II) ions [5, 19, 20]. On the other hand, LPS and Cu(II) are reported to increase the microglial production of mitochondrial ROS (mtROS), which can drive the production of proinflammatory cytokines and chemokines [21–24].

Therefore, the present study was aimed to determine (1) whether Cu(II) coordination enhances the effect of Aβ on microglial activation and the subsequent neurotoxicity, (2) whether this effect involves NF-κB and ROS, and (3) where these ROS are generated.

## Materials and methods

### Chemicals

Human Aβ_1–40_ or Aβ_1–42_, dihydroethidium (DHE), MitoTracker Green, and MitoSOX Red were purchased from Invitrogen (Carlsbad, CA, USA). Mouse anti-A11 or 6E10 antibodies were from Covance (San Diego, CA, USA). CuCl_2_, ZnCl_2_, cytosine arabinoside, pentoxifylline, aminoguanidine, N-acetyl-cysteine (NAC), apocynin, diphenylene iodonium (DPI), and LPS were from Sigma-Aldrich (St. Louis, MO, USA). BAY11-7082 and SC-514 were purchased from Merck (Darmstadt, Germany). Rabbit anti-inducible nitric oxide synthase (iNOS), IκB-α, phospho-IκB-α, p65, phospho-p65, β-actin, or microtubule-associated protein 2 (MAP2) antibodies were acquired from Cell Signaling Technology (Beverly, MA, USA). Mouse anti-CD11b antibody was purchased from Abcam (Cambridge, MA, USA).

### Preparation of Cu(II)-Aβ complex, fibrillar Aβ, and oligomeric Aβ

Lyophilized Aβ_1–40_ or Aβ_1–42_ (1.0 mg) was dissolved in 1 ml hexafluoroisopropanol (HFIP) for 2 h at room temperature. Then, HFIP was removed under a gentle stream of nitrogen gas. The lyophilized peptide was dissolved in dimethyl sulfoxide (DMSO) to obtain a 500 μM stock solution. To generate the Cu(II)-Aβ complex, Aβ stock solution was brought to the required concentrations in 20 mM Hepes buffer (containing 153 mM NaCl; pH 6.6) in the presence or absence of CuCl_2_ (with an equimolar Aβ:Cu(II) ratio). The reaction mixtures were incubated for 24 h at 37 °C. Similarly, the Zn(II)-Aβ complex was prepared with Aβ_1–40_ and ZnCl_2_ (with an equimolar Aβ:Zn(II) ratio). In the cellular experiments, the Cu(II)-Aβ or Zn(II)-Aβ complex was diluted with the culture media to appropriate concentrations.

Fibrillar Aβ was prepared by dissolving Aβ_1–40_ in ddH_2_O, which was followed by incubation at 37 °C for 7 days [5]. To prepare Aβ oligomers, Aβ_1–40_ was dissolved to 500 μM in HFIP and was diluted to 50 μM with ddH_2_O. After a 10- to 20-min incubation at room temperature, the samples were centrifuged at 14,000×*g* for 15 min. The supernatant was transferred to a new siliconized tube and subjected to a gentle stream of nitrogen gas for 5–10 min to evaporate the HFIP. The samples were then stirred at 500 rpm using a Teflon-coated micro stir bar for 48 h at 22 °C [25].

### Aggregation assay

To determine the Cu(II)-induced Aβ aggregation, the reaction mixtures containing 50 μM Aβ_1–40_ with or without 50 μM Cu(II) were incubated for 24 h at 37 °C; then, they were centrifuged at 13,000×*g* for 15 min to sediment aggregated proteins. The soluble peptide concentration in the supernatant was determined using Coomassie Plus (Bradford) Assay Reagent (Thermo, Rockford, IL, USA) [13, 26]. To quantify the amyloid fibrils, the thioflavin T fluorescence assay was applied [26]. After incubation, the samples containing Cu(II)-Aβ_1–40_, Aβ_1–40_, or fibrillar Aβ_1–40_ at 50 μM peptide concentration were diluted with 50 mM glycine-NaOH buffer (pH 8.5) containing 10 μM thioflavin T (Sigma-Aldrich) to a final volume of 100 μl. Fluorescence was monitored using a Varioskan Flash multimode reader (Thermo), with an excitation at 446 nm and emission at 490 nm.

### Dot blot

Five microliters of samples containing Cu(II)-Aβ (with equimolar or subequimolar Aβ_1–40_:Cu(II) ratios) or oligomeric Aβ_1–40_ at a 10 μM peptide concentration were applied to a nitrocellulose membrane (Millipore, Bedford, MA, USA). The membrane was air dried and then blocked with 10 % nonfat milk in Tris-buffered saline with Tween 20 (TBST) overnight at 4 °C. Following three 5-min washes, the membrane was incubated with A11 antibody (1:1000) for 1 h at room temperature. After washing, the blots were incubated with horseradish peroxidase-conjugated anti-rabbit IgG (1:10,000, Santa Cruz, Dallas, TX, USA) for 1 h at room temperature. The blots were detected with a chemiluminescence kit (Pierce, Rockford, IL, USA). Then, the same membrane was stripped and immunoblotted with 6E10 (1:1000), as described above.

### Microglial cell culture

The immortalized mouse microglial cell line BV-2 was routinely grown in Dulbecco’s modified Eagle’s medium (DMEM; Gibco, Grand Island, NY, USA) supplemented with 10 % fetal calf serum (FCS; Gibco), 100 U/ml penicillin, and 100 μg/ml streptomycin in a humidified atmosphere containing 5 % CO_2_ at 37 °C. Primary microglial cultures from newborn (24 h) Sprague-Dawley pups (Shanghai Laboratory Animal Center, Chinese Academy of Science, Shanghai, China) were prepared as previously described [21]. Briefly, cerebral cortex was dissected out and minced in cold DMEM. After a 20-min trypsinization (0.25 %), the cell suspension was centrifuged at 900×*g* for 10 min. The pelleted cells were resuspended and seeded in 75-cm^2^ tissue culture flasks containing DMEM supplemented with 10 % FCS. The mixed glial cultures were maintained at 37 °C in a humidified 5 % CO_2_ atmosphere, and the medium was changed every 48 h. Once confluent (12–14 days), the flasks were shaken (37 °C, 72 rpm) for 6 h. Microglial cells were harvested, centrifuged, and seeded onto 96-well plates at a density of 5 × 10^4^ cells/well.

To obtain conditioned media for treating neurons, primary microglia were first cultured in 96-well plates for 24 h in DMEM supplemented with 10 % FCS. Then, they were washed and changed to the Neurobasal medium with 2 % B27 supplement (Gibco) and cultured for 24 h in the presence or absence of Cu(II)-Aβ_1–40_, Aβ_1–40_ (preincubated at 37 °C for 24 h; if not mentioned, the preincubated Aβ_1–40_ was used in the following experiments), or Cu(II). The microglia-conditioned media were collected and briefly centrifuged; then, they were diluted with Neurobasal medium containing B27 to treat neurons.

### Primary hippocampal neuron culture

Hippocampi dissected from pups at postnatal day 1 were used to prepare neuronal cultures as previously described [27]. Briefly, pooled hippocampi were washed, triturated, and then treated with papain for 20 min. The mixture was centrifuged at 900×*g* for 5 min, and the precipitate was resuspended with MEM (Gibco) containing 10 % FCS and 5 % horse serum. The cells were seeded onto a 96-well plate at a density of 1 × 10^4^/well. The medium was changed to Neurobasal medium with B27 supplement 4 h after plating. To prevent the proliferation of nonneuronal cells, cytosine arabinoside (10 μM) was added to the cultures at the second day. Half of the medium was removed every 3 days and replaced with fresh medium. Neurons were cultured for 10–14 days for experiments.

### Cell viability assay

Microglial cells and hippocampal neurons were seeded onto 96-well plates and allowed to grow. When they reached the required confluence, the medium was changed to serum-free medium, and the cells were treated with Cu(II)-Aβ_1–40_, Aβ_1–40_, or Cu(II) for 24 h. The survival of cells was determined by incubating the cells with a cell counting kit-8 (CCK-8; 5 mg/ml; Dojindo, Kumamoto, Japan) at 37 °C for 2 h. The absorbance was measured at a wavelength of 450 nm. The cell viability of each treatment group was calculated relative to the control group that was treated with the medium. In another experiment, hippocampal neurons were treated with conditioned media from Cu(II)-Aβ_1–40_-stimulated microglia. The cell viability was determined 24 h later.

### Measurement of tumor necrosis factor-α (TNF-α) and nitric oxide production

The TNF-α concentration in the culture media of BV-2 or primary microglial cells was determined using an enzyme-linked immunosorbent assay kit (Pierce) according to the manufacturer’s instructions.

Griess reagent (Sigma-Aldrich) was used to measure the nitric oxide production. Briefly, 50 μl of medium from each culture well was placed in triplicate wells of a 96-well plate. An equal volume of Griess reagent was added to each well for 10 min. The optical densities were measured at 540 nm. The nitrite concentration was determined from a standard curve prepared by adding NaNO_2_ to DMEM.

### Hydrogen peroxide (H_2_O_2_) assay

The production of H_2_O_2_ in BV-2 cells was quantified by a fluorometric assay with Amplex Red reagent (Molecular Probes, Eugene, OR, USA) according to the manufacturer’s instructions. Briefly, the reaction mixtures in Krebs-Ringer phosphate buffer with glucose (KRPG; 145 mM NaCl, 5.7 mM Na_3_PO_4_, 4.86 mM KCl, 0.54 mM CaCl_2_, 1.22 mM MgSO_4_, 5.5 mM glucose, pH 7.35) containing 50 μM Amplex Red and 0.1 U/ml horseradish peroxidase were prewarmed at 37 °C for 10 min; then, 1 × 10^5^ cells resuspended in KRPG, and Cu(II)-Aβ_1–40_, Aβ_1–40_, Cu(II), or medium (for control group), were added. The absorbance at 560 nm was measured at different time points using a Varioskan Flash multimode reader (Thermo).

### Cytosolic and mitochondrial ROS detection

BV-2 cells were seeded in 3.5-cm cell culture dishes at a density of 1.5 × 10^5^/well. When the cells reached the required confluence, they were stimulated with Cu(II)-Aβ_1–40_, Aβ_1–40_, or Cu(II) for 1 h. Then, the cells were washed twice with PBS and labeled at 37 °C for 30 min with the following fluorescent probes: 0.1 μM DHE (oxidized by cytosolic ROS to fluorescent ethidium that binds nuclear DNA, Ex_518 nm_/Em_605 nm_), 200 nM MitoTracker Green (specifically labels mitochondria, Ex_490 nm_/Em_516 nm_), and 5 μM MitoSOX Red (specifically detects mitochondrial superoxide, Ex_510 nm_/Em_580 nm_). The fluorescence intensity was detected on a Nikon Eclipse 80i microscope (Melville, NY, USA).

### Immunofluorescence staining

Primary microglial cells or hippocampal neurons were fixed with 4 % paraformaldehyde for 1 h and blocked in 10 % BSA for 1 h at room temperature. Then, they were stained overnight at 4 °C with mouse anti-CD11b (1:200) or rabbit anti-MAP2 (1:50) antibodies. After incubation with an Alexa Fluor 488 donkey anti-mouse or anti-rabbit IgG secondary antibody (Invitrogen), cells were counterstained with 4′,6-diamidino-2-phenylindole (DAPI) and imaged by a Leica TCS SP2 AOBS confocal microscope (Leica, Wetzlar, Germany).

### Western blot

BV-2 cells were plated at a density of 1.5×10^5^ cells/ml into 6-well plates and allowed to grow. At different time points of Cu(II)-Aβ_1–40_ treatment, the cells were collected and lysed with a radioimmunoprecipitation assay (RIPA) buffer (50 mM Tris [pH 7.4], 150 mM NaCl, 1 % Triton X-100, 1 % sodium deoxycholate, 0.1 % sodium dodecyl sulfate [SDS], EDTA, sodium orthovanadate, sodium fluoride, and leupeptin) containing a protease inhibitor cocktail (Sigma-Aldrich). Equal amounts (30 μg) of proteins were loaded on a 10 % SDS-PAGE gel and transferred to polyvinylidene fluoride membranes. To avoid nonspecific binding, the membrane was blocked with 5 % nonfat milk in TBST for 1 h at room temperature and then incubated overnight at 4 °C with primary antibodies specific for iNOS, phospho-IκB-α, IκB-α, phospho-p65, p65, or β-actin at a 1:1000 dilution. The membrane was washed three times with TBST and incubated for 1 h with a horseradish peroxidase-conjugated secondary antibody (1:5000; Abcam). The bands were visualized using enhanced chemiluminescence (Pierce).

### Data analysis and statistics

Data are represented as the means ± SEM. Statistical analyses were performed using Student’s *t*-test and one-way or two-way analysis of variance (ANOVA) followed by Bonferroni post-test comparisons. Differences of *P* < 0.05 were considered statistically significant.

## Results

### Formation of the Cu(II)-Aβ complex

Incubation of Cu(II) with Aβ_1–40_ for 24 h at 37 °C resulted in a dramatically decreased protein content in the supernatant (*P* < 0.01 vs Aβ alone; Additional file 1: Figure S1A). The results of the thioflavin T (specifically detecting the β-sheet structure) fluorescence assay suggest that the conformation of Cu(II)-Aβ_1–40_ was different from Aβ_1–40_ or fibrillar Aβ_1–40_ (Additional file 1: Figure S1B). Those of dot blot analysis with anti-oligomer A11 antibody suggest that the nature of Cu(II)-Aβ_1–40_ was different from that of oligomeric Aβ_1–40_ (Additional file 1: Figure S1C). Collectively, these data indicate that Cu(II) interacts with Aβ, which leads to the formation of the Cu(II)-Aβ complex.

### Cu(II)-Aβ complex induces microglial activation

**Fig. 1:**
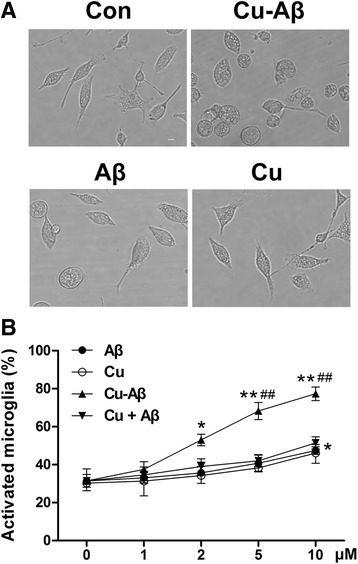
Cu(II)-Aβ complex triggers the activation of primary microglia. **a** The resting microglia (*con*) had branched processes, whereas microglia incubated with Cu(II)-Aβ_1–40_ (at 5 μΜ peptide concentration) for 24 h had retracted processes and a rounded morphology. Aβ_1–40_ and Cu(II) alone had no obvious effect on the morphological phenotype of microglia. Scale bar = 35 μm. **b** Effect of Cu(II)-Aβ on the fraction of microglia exhibiting the activated morphology. Microglia were treated with Cu(II)-Aβ_1–40_, Aβ_1–40_, Cu(II), or Cu(II) plus Aβ_1–40_ (Cu + Aβ; without preincubation) for 24 h. The amounts of microglia with activating morphology were counted, and the fractions of activated microglia were calculated. Data are expressed as the means ± SEM of at least three independent experiments. Significance was tested by one-way or two-way ANOVA followed by Bonferroni post-test comparisons. *P < 0.05, **P < 0.01 vs control (medium-treated); ^##^ *P* < 0.01 vs Aβ or Cu(II) alone

Using the Cu(II)-Aβ_1–40_ complex that we prepared, we compared its effect on microglial activation with Aβ_1–40_ or Cu(II) alone. The primary microglial cultures were uniformly immunopositive for CD11b (a microglia marker) and contained approximately 95 % microglial cells (Additional file 2: Figure S2). The amounts of microglia exhibiting activating morphology after Cu(II)-Aβ_1–40_ treatment were counted with the photographs (magnification: ×100) taken with an inverted microscopy (EVOS FI; AMG, WA, USA) by a skilled experimenter who was blinded to the treatments, and the fractions of activated microglia were calculated. Cu(II)-Aβ_1–40_ treatment caused a morphological transformation of microglia from the resting to active phenotype (Fig. 1a). The increase in the number of activated microglia was significant at 2–10 μM peptide concentrations of Cu(II)-Aβ_1–40_ (*P* < 0.05 or 0.01 vs control; Fig. 1b). Whereas, the same concentrations (except 10 μM) of Aβ_1–40_ and Cu(II) had no obvious effect on the morphological phenotype of microglia and the number of activated microglia (Fig. 1a, b). Treatment with Cu(II) plus Aβ_1–40_ (Cu + Aβ; without preincubation) did not alter the number of activated microglia (Fig. 1b). Microglial cell death was negligible when the peptide concentrations of Cu(II)-Aβ_1–40_ were below 10 μM (data not shown). We also examined the effect of Cu-Aβ_1–42_ and found that Cu-Aβ_1–42_ at a 5 μM peptide concentration elicited an increased fraction of activated microglia (74.3 ± 4.6 %; *P* < 0.01 vs control [32.3 ± 3.4 %], Aβ_1–42_ [44.2± 3.8 %], or Cu(II) [38.9 ± 3.6 %]; *n* = 3).

### Cu(II)-Aβ complex induces TNF-α production in microglial cells

**Fig. 2:**
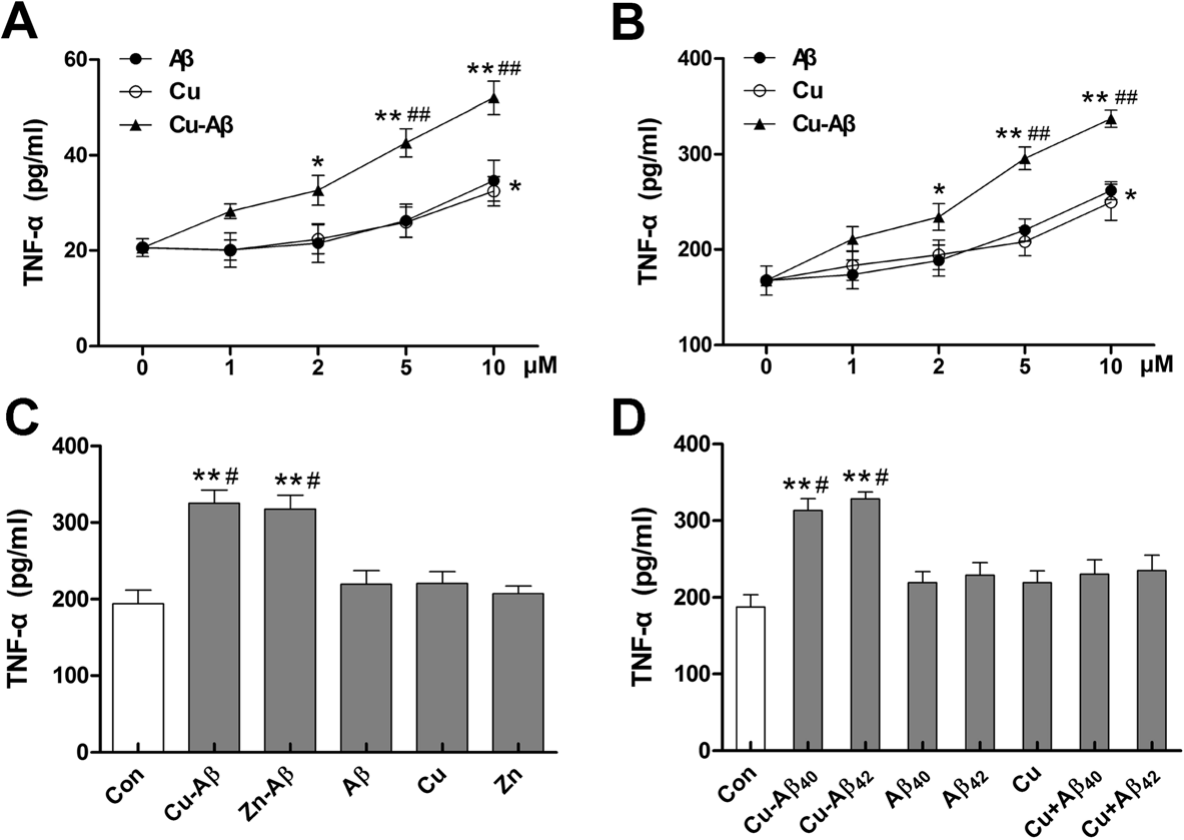
TNF-α concentration in the media from microglial cells. Primary (**a**) and BV-2 (**b**) microglial cells were stimulated with Cu(II)-Aβ_1–40_ (at 1–10 μΜ peptide concentrations), Aβ_1–40_, or Cu(II) for 24 h. **c** BV-2 cells were stimulated with Cu(II)-Aβ_1–40_ or Zn(II)-Aβ_1–40_ (at 5 μΜ peptide concentration), Aβ_1–40_, Cu(II), or Zn(II) for 24 h. **d** BV-2 cells were stimulated with Cu(II)-Aβ_1–40_ or Cu(II)-Aβ_1–42_ (at 5 μΜ peptide concentration), Aβ_1–40_ or Aβ_1–42_, Cu(II), or Cu(II) plus Aβ_1–40_ or Aβ_1–42_ (Cu + Aβ_40_ or Cu + Aβ_42_; without preincubation) for 24 h. Data are expressed as the means ± SEM of at least three independent experiments. Significance was tested by one-way or two-way ANOVA followed by Bonferroni post-test comparisons. *P < 0.05, **P < 0.01 vs control; ^#^ *P* < 0.05, ^##^ *P* < 0.01 vs Aβ, Cu(II), or Zn(II) alone

TNF-α concentrations in the media of primary microglial cultures and BV-2 cells were increased in a dose-dependent manner after Cu(II)-Aβ_1–40_ stimulation, and TNF-α production induced by 5–10 μM of Cu(II)-Aβ_1–40_ was more robust than that induced by Aβ_1–40_ or Cu(II) alone (*P* < 0.01); Aβ_1–40_ and Cu(II) at 1–5 μM had no obvious effect on the TNF-α content; while at 10 μM, they induced significantly increased TNF-α production (*P* < 0.05 vs control; Fig. 2a, b). Zn(II) can also bind to Aβ to form Zn(II)-Aβ complex [9]. So, we also examined the effect of Zn(II)-Aβ_1–40_ on microglial release of TNF-α. We found that Zn(II)-Aβ_1–40_ caused similar TNF-α production to that induced by Cu(II)-Aβ_1–40_ in BV-2 cells (Fig. 2c). In addition, Cu(II)-Aβ_1–42_ also elicited microglial release of TNF-α in BV-2 cells (Fig. 2d). Treatment with addition of Cu(II) and Aβ without preincubation had little effect on TNF-α production in BV-2 cells (Fig. 2d).

### Cu(II)-Aβ complex induces nitric oxide production in microglial cells

**Fig. 3:**
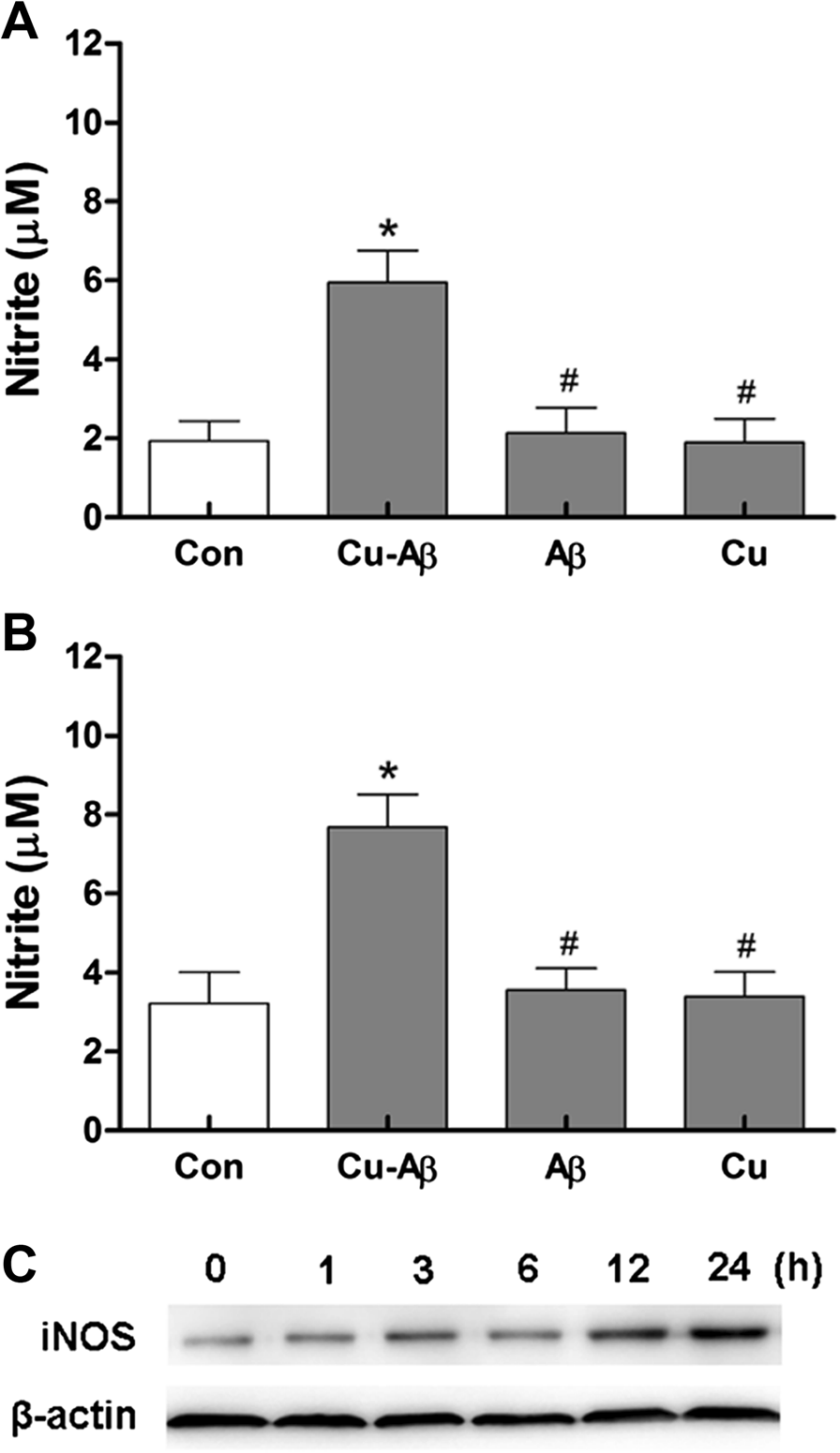
Nitric oxide concentration in the media from primary (**a**) and BV-2 (**b**) microglial cells. Microglial cells were stimulated with Cu(II)-Aβ_1–40_ (5 μΜ peptide), Aβ_1–40_, or Cu(II) for 24 h. Data are expressed as the means ± SEM of at least three independent experiments. Significance was tested by one-way ANOVA followed by Bonferroni post-test comparisons. *P < 0.05 vs control; ^#^ *P* < 0.05 vs Cu-Aβ. **c** Western blot analysis of iNOS expression in BV-2 cells after Cu(II)-Aβ stimulation

Next, we examined the nitric oxide production in microglial cells. Cu(II)-Aβ_1–40_ treatment led to significantly increased nitric oxide content in the media from both primary and BV-2 microglial cells, as evaluated by measuring the concentration of nitrite (NO_2_^−^), its stable metabolite; whereas, Aβ_1–40_ or Cu(II) alone had no obvious effect on the nitrite content (Fig. 3a, b). In parallel, Western blot analysis with BV-2 cells revealed an induction of iNOS expression in response to Cu(II)-Aβ_1–40_ stimulation, which was prominent at 24 h (Fig. 3c). Aβ_1–40_ or Cu(II) alone had no obvious effect on the iNOS expression (Additional file 3: Figure S3).

### Cu(II)-Aβ complex causes indirect neuronal damage via microglia

**Fig. 4:**
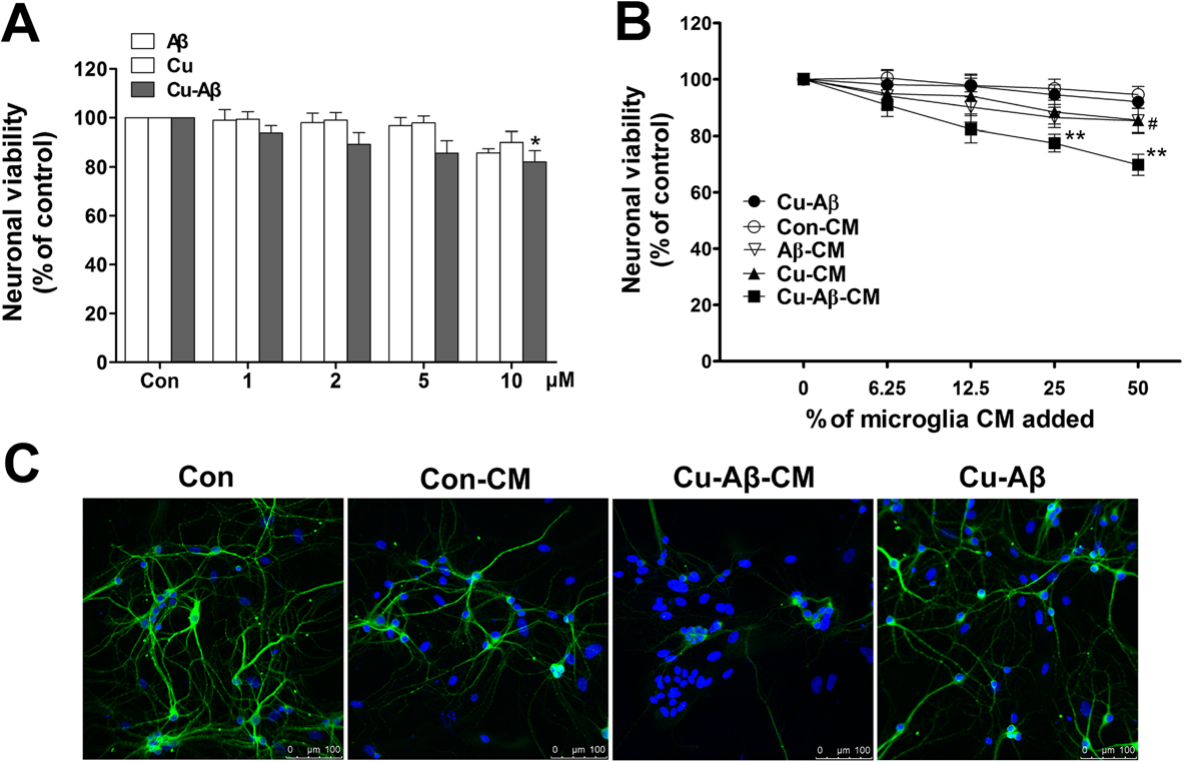
Cu(II)-Aβ complex causes indirect, microglia-mediated neurotoxicity. **a** Cell viability of hippocampal neurons treated with Cu(II)-Aβ_1–40_ (1–10 μΜ peptide), Aβ_1–40_, or Cu(II) for 24 h. **b** Conditioned media (*CM*) from microglia stimulated with Cu(II)-Aβ_1–40_ (5 μΜ peptide, 24 h) caused neurotoxicity. Hippocampal neurons were treated with five types of media diluted into the neuronal culture media at indicated percentiles for 24 h. They are (*1*) media with direct addition of Cu(II)-Aβ_1–40_ (5 μM peptide), (*2*) media previously conditioned by unstimulated microglia (*con-CM*), (*3*) media previously conditioned by Aβ_1–40_-stimulated microglia (*Aβ-CM*), (*4*) media previously conditioned by Cu(II)-stimulated microglia (*Cu-CM*), and (*5*) media previously conditioned by Cu(II)-Aβ_1–40_-stimulated microglia (*Cu-Aβ-CM*). Data are expressed as the means ± SEM of at least three independent experiments. Significance was tested by one-way or two-way ANOVA followed by Bonferroni post-test comparisons. *P < 0.05, **P < 0.01 vs control (**a**) or con-CM (**b**); ^#^ *P* < 0.05 vs Aβ-Cu-CM. **c** Cu(II)-Aβ_1–40_ caused indirect, microglia-mediated damage to neuronal dendrites. All CM used were diluted at 50 % into the neuronal culture media. The control group was treated with solvent only. Neuronal dendrites were observed by immunostaining for MAP2 (*green*)

We first examined whether Cu(II)-Aβ_1–40_ causes direct neuronal damage at doses required for activating microglia. Cu(II)-Aβ_1–40_, at 1–5 μM peptide concentrations, did not cause obvious neuronal death (Fig. 4a). Next, we activated cultured primary microglia using a subneurotoxic concentration (5 μM) of Cu(II)-Aβ_1–40_ and transferred the microglia-conditioned media (Cu-Aβ-CM) to neuronal cultures. In the meantime, we treated neurons with conditioned media derived from microglia treated with solvent (con-CM, used as negative controls), Aβ_1–40_ (5 μM; Aβ-CM), Cu(II) (5 μM; Cu-CM), or media with Cu(II)-Aβ_1–40_ but without being conditioned by microglia (Cu-Aβ). Cu-Aβ-CM reduced neuronal survival in a dose-dependent manner (*P* < 0.01 vs con-CM at 25–50 % of media). Whereas, the equivalent amount of con-CM, Aβ-CM, Cu-CM, or Cu-Aβ directly added to the media without being conditioned by microglia did not reduce the neuronal viability (Fig. 4b). We used Triton X-100 (at 100 μM), which results in the necrotic death of the cells [28], as a cell death control for the cell viability assay. We found that the viability of Triton X-treated neurons was less than 10 % (9.6 ± 2.1 %; *n* = 3).

Dendritic damage is an important pathological process in neuronal loss in AD. To further confirm the indirect neurotoxicity produced by Cu(II)-Aβ_1–40_, we stained the neuronal dendrites with an antibody against MAP2, a dendritic marker. We found that neurons treated with Cu-Aβ-CM had markedly dendritic damage than those treated with con-CM (Fig. 4c). Aβ_1–40_ or Cu(II) alone had no obvious effect on the MAP2 immunofluorescence (data not shown).

### TNF-α and nitric oxide are involved in Cu(II)-Aβ complex-caused, microglia-mediated neurotoxicity

**Fig. 5:**
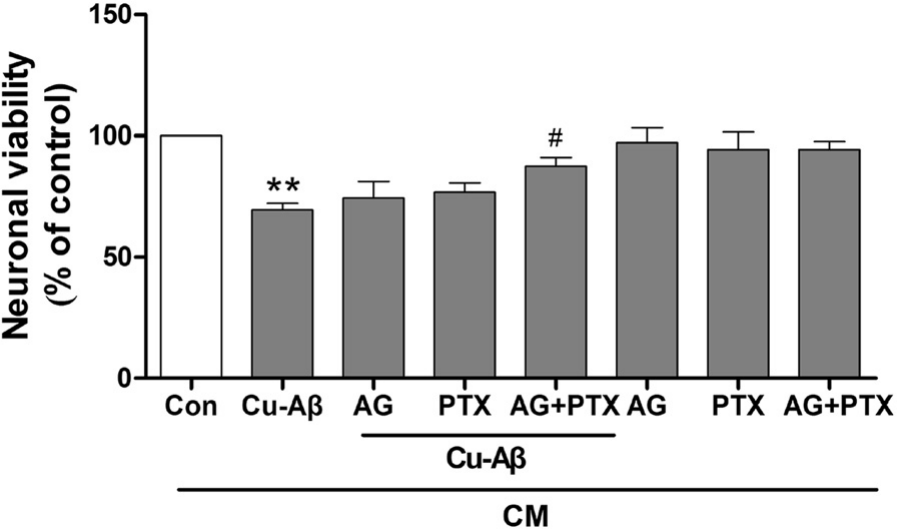
TNF-α and nitric oxide are involved in Cu(II)-Aβ complex-caused, microglia-mediated neurotoxicity. Cell viability of hippocampal neurons treated with conditioned media (*CM*, at 50 % of neuronal culture media) from primary microglia stimulated with Cu(II)-Aβ_1–40_ (5 μΜ peptide) in the presence or absence of pentoxifylline (*PTX*, 1 mM) and/or aminoguanidine (*AG*, 200 μM). Data are expressed as the means ± SEM of at least three independent experiments. Significance was tested by one-way ANOVA followed by Bonferroni post-test comparisons. **P < 0.01 vs con-CM; ^#^ *P* < 0.05 vs Cu-Aβ-CM

Subsequently, we examined the effect of inhibiting microglial production of TNF-α and nitric oxide with pentoxifylline, a nonselective phosphodiesterase inhibitor that blocks the release of TNF-α from microglia and does not affect nitrite accumulation [29], and aminoguanidine, an iNOS inhibitor. We found that the conditioned media from microglia treated with pentoxifylline plus aminoguanidine (but not pentoxifylline or aminoguanidine alone) in the presence of Cu(II)-Aβ_1–40_ induced less neuronal death than the conditioned media from microglia treated with Cu(II)-Aβ_1–40_ (*P* < 0.05; Fig. 5). Pentoxifylline or aminoguanidine did not affect neuronal survival (data not shown).

### NF-κB is required for Cu(II)-Aβ complex-induced microglial activation

**Fig. 6:**
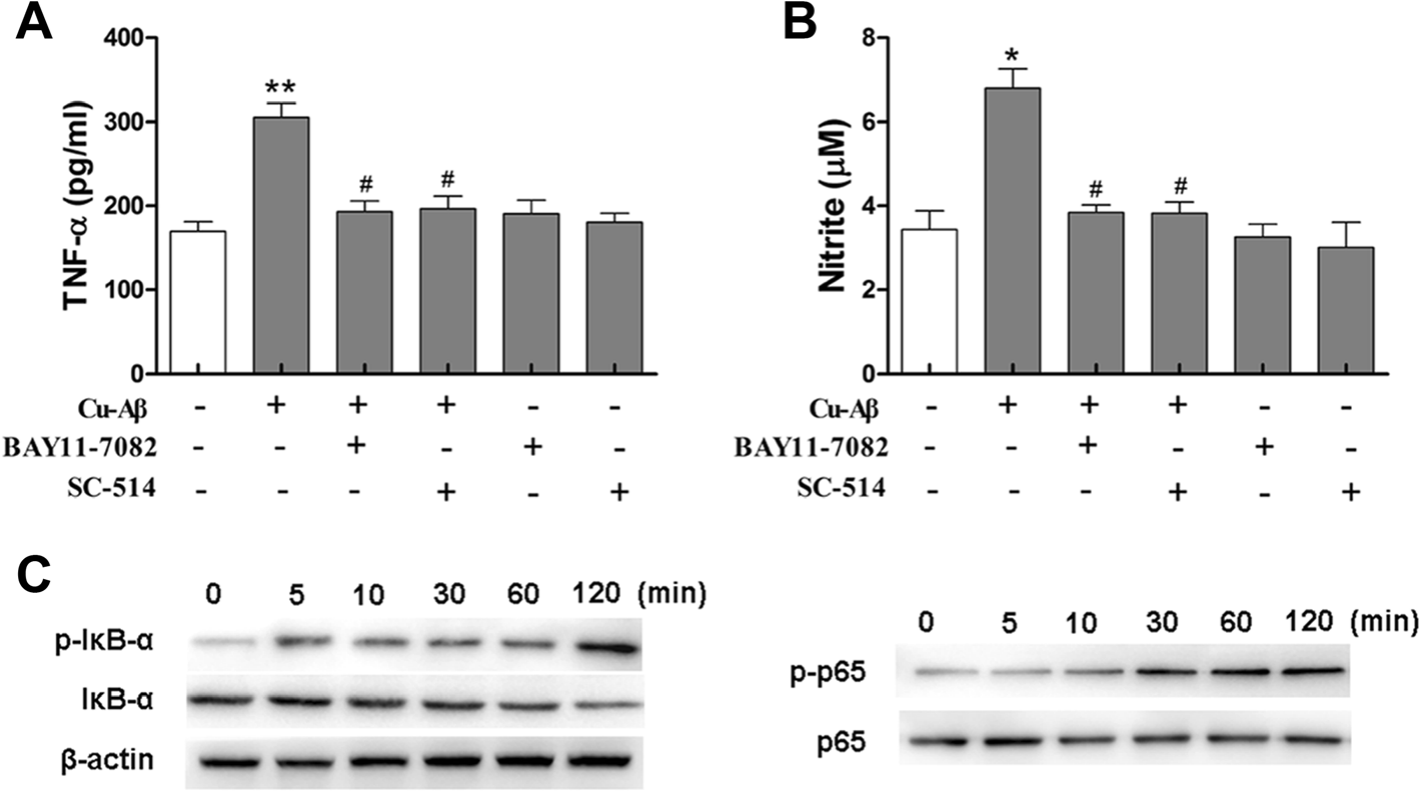
Cu(II)-Aβ complex-induced microglial activation depends on NF-κB. **a**, **b** BAY11-7082 and SC-514 inhibited Cu(II)-Aβ_1–40_-induced release of TNF-α or nitric oxide. BV-2 cells were pretreated with BAY11-7082 (10 μΜ) or SC-514 (50 μΜ) for 1 h prior to 24-h stimulation with Cu(II)-Aβ_1–40_ (5 μΜ peptide). Data are expressed as the means ± SEM of at least three independent experiments. Significance was tested by one-way ANOVA followed by Bonferroni post-test comparisons. *P < 0.05, **P < 0.01 vs control; ^#^ *P* < 0.05 vs Cu-Aβ. **c** The phosphorylation and degradation of IкB-α and phosphorylation of p65 in BV-2 cells stimulated with Cu(II)-Aβ_1–40_. The cells were treated with Cu(II)-Aβ for indicated time intervals

NF-κB activation is critical for cellular inflammatory responses, such as microglial activation [16, 30]. Therefore, we investigated whether NF-кB is involved in Cu(II)-Aβ_1–40_-primed microglial activation. Both BAY11-7082 and SC-514, the inhibitors for IκB-α phosphorylation and IκB kinase-2 (IKK-2), respectively, abrogated Cu(II)-Aβ_1–40_-induced TNF-α or nitric oxide production in BV-2 cells (Fig. 6a, b). In parallel, Western blot analysis revealed that Cu(II)-Aβ_1–40_ treatment induced phosphorylation and degradation of IκB-α protein as well as phosphorylation of NF-кB p65 (Fig. 6c).

### Cu(II)-Aβ complex induces NOX-independent extracellular H_2_O_2_ release

**Fig. 7:**
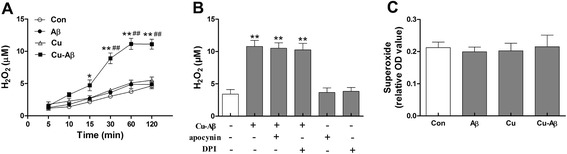
Cu(II)-Aβ complex induces NADPH oxidase-independent extracellular H_2_O_2_ release. **a** H_2_O_2_ concentration in the media from BV-2 cells after stimulation with Cu(II)-Aβ_1–40_ (5 μM peptide), Aβ_1–40_, or Cu(II). **b** H_2_O_2_ concentration in the media from BV-2 cells treated with Cu(II)-Aβ_1–40_ (for 1 h) in the presence or absence of apocynin (100 μΜ) or DPI (10 μΜ). **c** Cu(II)-Aβ_1–40_ stimulation (for 1 h) had no obvious effect on extracellular superoxide release. Data are expressed as the means ± SEM of at least three independent experiments. Significance was tested by one-way or two-way ANOVA followed by Bonferroni post-test comparisons. *P < 0.05, **P < 0.01 vs control; ^##^ *P* < 0.01 vs Aβ or Cu(II) alone

ROS, such as H_2_O_2,_ are critical for regulating the glial inflammatory response to various stimuli [20]. We detected H_2_O_2_ release induced by Cu(II)-Aβ_1–40_ using a fluorometric assay with Amplex Red. Cu(II)-Aβ_1–40_ stimulation led to a rapid accumulation of H_2_O_2_ in the culture media of BV-2 cells, while Aβ_1–40_ or Cu(II) alone had no obvious effect on H_2_O_2_ production (Fig. 7a). We then used two NOX inhibitors, apocynin and DPI, to determine whether the H_2_O_2_ is derived from NOX. Neither apocynin nor DPI affected H_2_O_2_ release elicited by Cu(II)-Aβ_1–40_ (Fig. 7b). In addition, Cu(II)-Aβ_1–40_ was unable to evoke extracellular superoxide release (Fig. 7c).

### Cu(II)-Aβ complex induces mitochondrial superoxide production

**Fig. 8:**
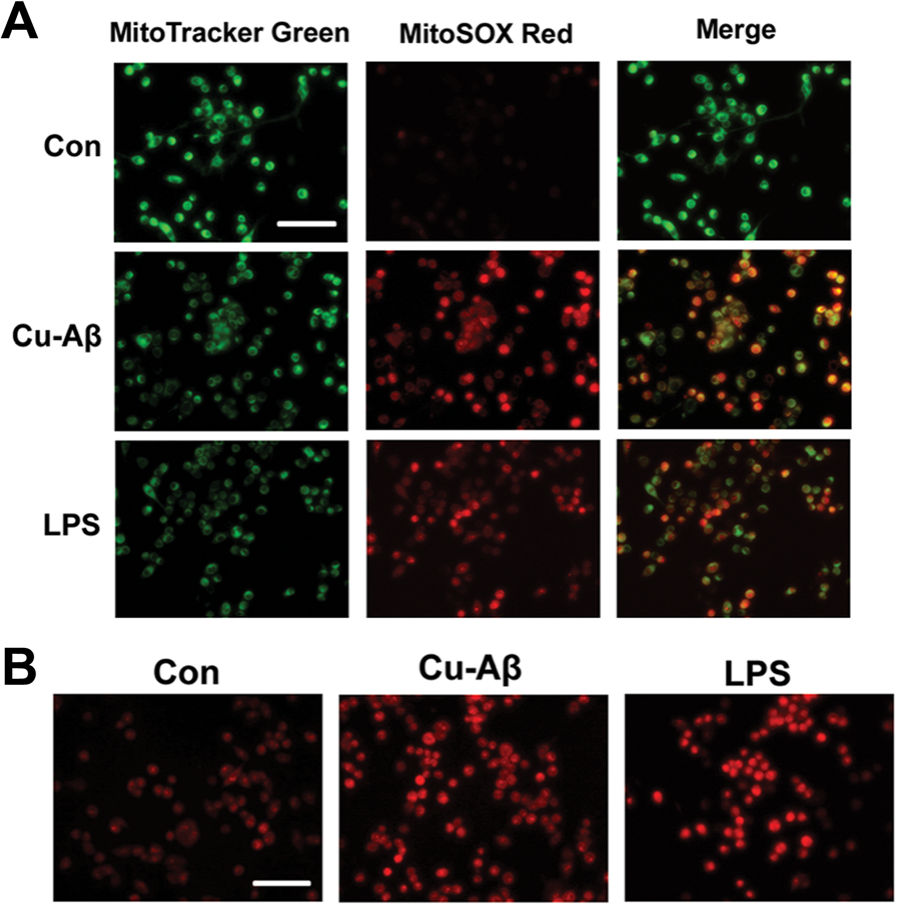
Cu(II)-Aβ complex induces mitochondrial superoxide production in BV-2 cells. **a** Overlay images of cells stained with MitoSOX Red (*red*, detects mitochondrial ROS) and MitoTracker Green (*green*, localizes to mitochondria) after Cu(II)-Aβ_1–40_ (5 μM peptide) stimulation for 1 h. **b** Cytosolic ROS were stained with a fluorescent probe dihydroethidium after Cu(II)-Aβ_1–40_ stimulation for 1 h. LPS (1 μg/ml) was used as positive control. *Bar* = 100 μm

Mitochondria are another intracellular source of ROS apart from NOX [31, 32]. Therefore, we detected mtROS production using MitoSOX, a fluorogenic dye that can be oxidized by superoxide and used as a mitochondrial superoxide indicator of live cells. Overlay images of cells labeled with MitoSOX Red and MitoTracker Green indicate that mitochondria are the primary site of ROS production in Cu(II)-Aβ_1–40_-stimulated BV-2 cells (Fig. 8a). Next, we labeled the cytosolic ROS with DHE and noted a significant increase in DHE fluorescence in cells stimulated with Cu(II)-Aβ_1–40_ (Fig. 8b). Aβ_1–40_ or Cu(II) at 5 μM had no obvious effect on the cytosolic or mitochondrial superoxide production (data not shown).

### ROS are involved in Cu(II)-Aβ complex-induced microglial activation and the indirect neurotoxicity

**Fig. 9:**
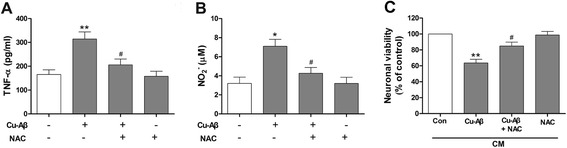
Effects of NAC on Cu(II)-Aβ complex-induced microglial activation and the indirect neurotoxicity. TNF-α (**a**) and nitric oxide (**b**) concentration in the media from BV-2 cells treated with Cu(II)-Aβ_1–40_ (5 μΜ peptide) for 24 h with or without NAC (1 mM). **c** Cell viability of hippocampal neurons treated with conditioned media (*CM*, at 50 % of neuronal culture media) from primary microglia stimulated with Cu(II)-Aβ_1–40_ in the presence or absence of NAC. Data are expressed as the means ± SEM of at least three independent experiments. Significance was tested by one-way ANOVA followed by Bonferroni post-test comparisons. *P < 0.05, **P < 0.01 vs control (**a**, **b**) or con-CM (**c**); ^#^ *P* < 0.05 vs Cu-Aβ (**a**, **b**) or Cu-Aβ-CM (**c**)

We treated BV-2 cells with Cu(II)-Aβ_1–40_ in the presence of NAC, a ROS scavenger. NAC inhibited the Cu(II)-Aβ_1–40_-induced microglial release of TNF-α and nitric oxide (Fig. 9a, b). Moreover, the conditioned media from primary microglia incubated with NAC in the presence of Cu(II)-Aβ_1–40_ failed to cause obvious neuronal death (Fig. 9c). NAC had no effect on neuronal survival (data not shown).

## Discussion

Our data show for the first time that Cu(II) enhances the effect of Aβ on microglial activation and the subsequent neurotoxicity. This activating effect is NF-κB-dependent and involves NOX-independent, mitochondria-derived ROS.

Cu(II) is suggested to bind monomeric Aβ peptides (predominantly with 1:1 stoichiometry) with high affinity, leading to the formation of Cu(II)-Aβ complex [7–9]. In the present study, the results of an assay of Aβ aggregation by sedimentation, thioflavin T fluorescence assay, and dot blot analysis indicate the formation of Cu(II)-Aβ complex, whose conformation or nature differs from fibrillar or oligomeric Aβ and monomeric Aβ.

In the following experiments, we found that Cu(II)-Aβ induced an activating morphological phenotype of microglia and microglial release of TNF-α and nitric oxide. The increase in nitric oxide production was accompanied by an increase in iNOS expression. Moreover, a subneurotoxic concentration of Cu(II)-Aβ produced an indirect, microglia-mediated neurotoxicity, as demonstrated by the impaired neuronal viability and dendritic damage. Importantly, Aβ or Cu(II) alone induced neither the microglial release of soluble neurotoxic factors nor the microglia-mediated neurotoxicity. Therefore, it is likely that the interactions between Cu(II) and Aβ lead to enhancement of the Aβ effect on microglial activation. In contrast to the observations that either neutralization of TNF-α or inhibition of iNOS is sufficient to prevent neurotoxicity produced by aggregated Aβ-activated microglia [29, 33], we found that repression of microglial release of both TNF-α and nitric oxide, but not of TNF-α or nitric oxide alone, was protective for hippocampal neurons. These data indicate that TNF-α and nitric oxide generated by Cu(II)-Aβ-stimulated microglia need to act synergistically to elicit neuronal damage.

NF-κB is an important regulator of the gene expression of proinflammatory mediators. The inhibitory IκB-α can be phosphorylated by activated IKK and then degraded, leading to phosphorylation and translocation of p65 and activation of gene transcription. In the present study, the inhibitors of IκB-α phosphorylation and IKK-2 prevented the Cu(II)-Aβ-induced microglial release of TNF-α and nitric oxide, suggesting that the actions of Cu(II)-Aβ depend on NF-кB. Cu(II)-Aβ stimulation also induced phosphorylation and degradation of IкB-α as well as phosphorylation of p65, further confirming the involvement of NF-кB.

ROS are critical for microglial activation [20, 34]. We found that Cu(II)-Aβ (but not Aβ or Cu(II) alone) triggered extracellular H_2_O_2_ accumulation. NOX, an enzyme that catalyzes the production of superoxide, is a producer of extracellular and intracellular ROS. We found that the NOX inhibitors did not affect Cu(II)-Aβ-induced H_2_O_2_ production. Furthermore, Cu(II)-Aβ was unable to evoke superoxide release. These data indicate that Cu(II)-Aβ-elicited extracellular H_2_O_2_ is not from NOX.

Mitochondria serve as another major source of ROS in most cells [31, 32]. Mitochondrion-generated superoxide, which does not cross the inner membrane, can be converted to membrane-permeable H_2_O_2_ by matrix Mn-superoxide dismutase, and H_2_O_2_ can diffuse to the cytosol and extracellular space (thus can be detected in the culture media) [35]. We observed an accumulation of mtROS in BV-2 cells stimulated with Cu(II)-Aβ. Given the finding that Cu(II)-Aβ-elicited extracellular H_2_O_2_ is not from NOX, it is likely that H_2_O_2_ originates in the mitochondria. In the meantime, cytosolic ROS accumulated in cells stimulated with Cu(II)-Aβ, indicating that some mtROS were diffused to cytosol. There is evidence that mtROS can diffuse to cytosol to signal NF-кB activation and proinflammatory cytokine expression [17, 22]. Therefore, our results strongly suggest that mtROS are involved in Cu(II)-Aβ-triggered microglial activation. Our findings that H_2_O_2_ release due to Cu(II)-Aβ stimulation is NOX-independent seem to be in contrast to those that fibrillar Aβ, LPS, or Zn(II) evokes NOX-dependent ROS production [5, 19, 20]. However, we noticed that, similar to the recent findings of Park et al. [23] and Voloboueva et al. [24], LPS elicited robust mtROS production. Therefore, microglial activation upon inflammatory stimulations may involve both NOX- and mitochondrion-derived ROS.

Noteworthy, we found that NAC (a ROS scavenger that readily passes into cells) inhibited the Cu(II)-Aβ-elicited microglial release of TNF-α and nitric oxide as well as microglia-mediated neuronal death. These results suggest that mtROS production precedes the production of soluble neurotoxic factors. Oxidative stress, an early event in AD pathogenesis, plays a critical role in neuronal dysfunction and death in this disease [36, 37]. Studies with murine models provide supportive evidence that administration of NAC blocks oxidative damage in AD [38, 39]. Clinical trials also show that NAC treatment is potentially beneficial to AD patients [38–41]. Our findings that NAC ameliorates neuronal injury mediated by Cu(II)-Aβ-activated microglia may further warrant the use of NAC in AD therapy.

In our work, preincubated (37 °C for 24 h) Aβ alone (up to 5 μM) had little effect on microglial activation. We noticed that the structure of these Aβ peptides is neither oligomeric nor fibrillar, which may be one explanation for this observation. Aβ can be taken up by microglia [42]. So, it is possible that Cu(II)-Aβ is taken up by microglial cells to induce mtROS production and that the difference in potency for microglial activation between Cu(II)-Aβ and Aβ is due to uptake difference.

The role of microglia in the pathogenesis of AD might be switched from a beneficially phagocytic effect to a proinflammatory cytotoxic role (with the classic M1 phenotype) with the age-dependent accumulation of extracellular Aβ aggregates [43]. At the early stage of AD, microglia might play a modulatory role via phagocytosis of Aβ plaques. In addition, microglia may exert protective functions through intracellular Cu(II) sequestration, which may prevent further plaque formation [44].

Interestingly, recent evidence shows that Cu(I), which also promotes Aβ aggregation [45], may polarize inflamed microglial populations from the neurotoxic (M1) phenotype to the neuroprotective (M2) phenotype via inhibition of nitric oxide production [46]. This observation implicates that the valence state of copper may be important in the regulation of microglial phenotype. The microglial responses to Cu(I)-Aβ and Cu(II)-Aβ and the subsequent neurotoxicity might be different as a result of their different redox capability.

Taken together, the present study identifies Cu(II) as a cofactor that promotes the effect of Aβ on microglial activation and the subsequent neurotoxicity. Moreover, the Cu(II)-Aβ-triggered microglial activation may involve NF-кB activation and mtROS production. These findings support the notion that metal ion-induced Aβ aggregation might be important in microglia-mediated neuroinflammation and might provide a novel anti-inflammatory strategy for AD.

## Additional files

Additional file 1: Figure S1. Formation of the Cu(II)-Aβ complex. (A) Peptide concentration in the supernatant of reaction mixtures containing Aβ_1–40_ (50 μM) with or without Cu(II) (50 μM). (B) Effect of Cu(II) on the formation of Aβ fibrils. The amyloid fibril formation of Aβ_1–40_ in the presence of Cu(II) (50 μM) was determined using the thioflavin T (ThT) fluorescence assay. Fibrillar Aβ_1–40_ (Aβf, 50 μM) was used as a positive conference. Data are expressed as the means ± SEM of three independent experiments. Significance was tested by Student’s *t*-test. ***P* < 0.01 vs Aβ (A) or Aβf (B); ^#^ *P* < 0.05 vs Aβ (B). (C) Dot blot analysis of Aβ_1–40_ and Cu(II)-Aβ_1–40_ (with 1:1 or 1:2 of Aβ:Cu(II) molar ratios) at a peptide concentration of 10 μM. Aβ_1–40_ was incubated in 20 mM Hepes buffer (containing 153 mM NaCl; pH 6.6) in the presence or absence of Cu(II) for 24 h at 37 °C. Oligomeric Aβ_1–40_ (Aβo) was used as a positive control. Additional file 2: Figure S2. Characterization of the microglial culture purity. Microglial cultures were immunostained with the microglial marker CD11b. Scale bar = 300 μm. Additional file 3: Figure S3. Western blot analysis of iNOS expression in BV-2 cells. (A) Representative Western blots of iNOS in BV-2 cells after Cu(II)-Aβ_1–40_ (at 5 μΜ peptide concentration), Cu(II), or Aβ_1–40_ stimulation (for 24 h). (B) Densitometric analysis of iNOS/β-actin ratio normalized to saline control. *n* = 3 experiments. ***P* < 0.01 vs con; ^#^ *P* < 0.05 vs Cu-Aβ.

## Abbreviations

AD: Alzheimer’s disease
Aβ: amyloid-β
CCK-8: Cell counting kit-8
CM: Conditioned medium
DMEM: Dulbecco’s modified Eagle’s medium
DPI: Diphenylene iodonium
FCS: Fetal calf serum
HFIP: Hexafluoroisopropanol
IKK-2: IκB kinase-2
iNOS: Inducible nitric oxide synthase
KRPG: Krebs-Ringer phosphate buffer with glucose
mtROS: mitochondrial ROS
NAC: N-acetyl-cysteine
NF-кB: nuclear factor-кB
NOX: NADPH oxidase
ROS: Reactive oxygen species
TBST: Tris-buffered saline with Tween 20
TNF-α: tumor necrosis factor-α
