# Supplementary material for: Cu(II) enhances the effect of Alzheimer’s amyloid-β peptide on microglial activation
Source: J Neuroinflammation. 2015 Jun 24;12:122. doi: 10.1186/s12974-015-0343-3 (PMC4490619; doi:10.1186/s12974-015-0343-3)
Supplement: Additional file 1: Figure S1. — Formation of the Cu(II)-Aβ complex. (A) Peptide concentration in the supernatant of reaction mixtures containing Aβ1–40 (50 μM) with or without Cu(II) (50 μM). (B) Effect of Cu(II) on the formation of Aβ fibrils. The amyloid fibril formation of Aβ1–40 in the presence of Cu(II) (50 μM) was determined using the thioflavin T (ThT) fluorescence assay. Fibrillar Aβ1–40 (Aβf, 50 μM) was used as a positive conference. Data are expressed as the means ± SEM of three independent experiments. Significance was tested by Student’s t-test. **P < 0.01 vs Aβ (A) or Aβf (B); # P < 0.05 vs Aβ (B). (C) Dot blot analysis of Aβ1–40 and Cu(II)-Aβ1–40 (with 1:1 or 1:2 of Aβ:Cu(II) molar ratios) at a peptide concentration of 10 μM. Aβ1–40 was incubated in 20 mM Hepes buffer (containing 153 mM NaCl; pH 6.6) in the presence or absence of Cu(II) for 24 h at 37 °C. Oligomeric Aβ1–40 (Aβo) was used as a positive control. [file 12974_2015_343_MOESM1_ESM.pdf]

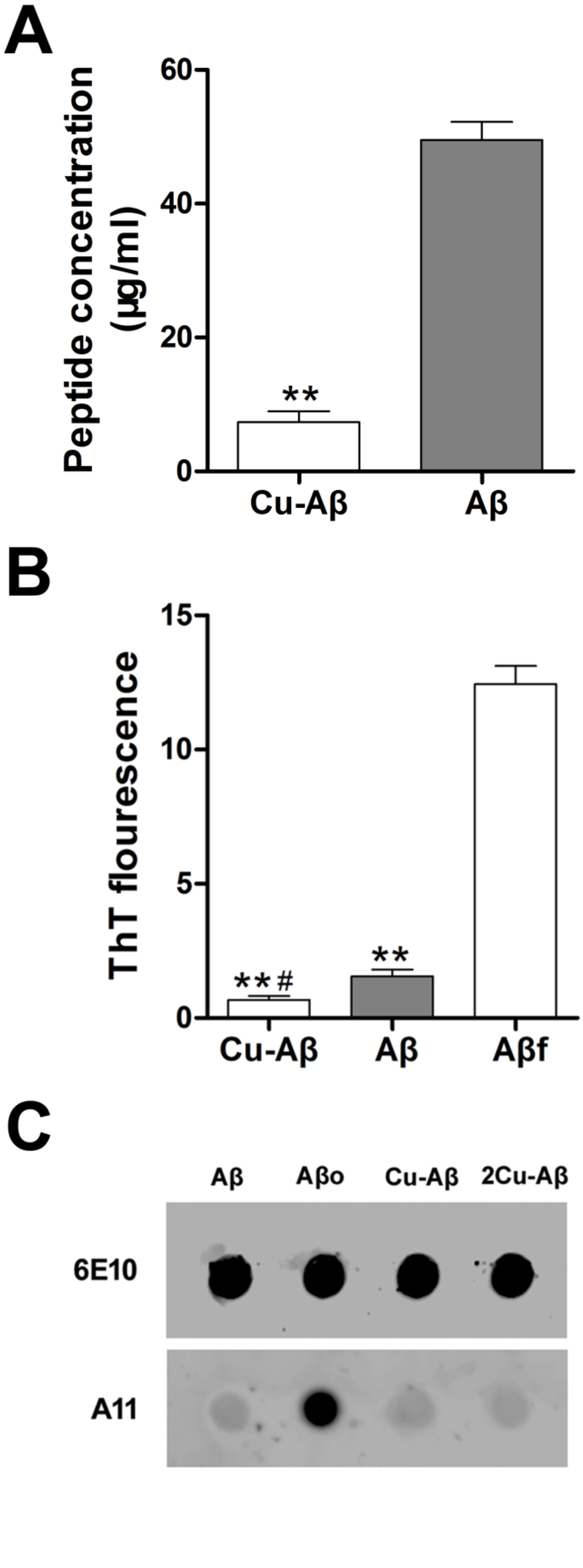

Additional file 1: Figure S1

Formation of the Cu(II)-Aβ complex. (A) Peptide concentration in the supernatant of

reaction mixtures containing Aβ_{1-40} (50 μM) with or without Cu(II) (50 μM). (B) Effect of Cu(II) on the formation of Aβ fibrils. The amyloid fibril formation of Aβ_{1-40} in the presence of Cu(II) (50 μM) was determined using the thioflavin T (ThT) fluorescence assay. Fibrillar Aβ_{1-40} (Aβf, 50 μM) was used as a positive conference. Data are expressed as the means ± SEM of three independent experiments. Significance was tested by Student's *t* test. $^{**}P < 0.01$ vs Aβ (A) or Aβf (B); $^{\#}P < 0.05$ vs Aβ (B). (C) Dot blot analysis of Aβ_{1-40} and Cu(II)-Aβ_{1-40} (with 1:1 or 1:2 of Aβ: Cu(II) molar ratios) at a peptide concentration of 10 μM. Aβ_{1-40} was incubated in 20 mM Hepes buffer (containing 153 mM NaCl; pH 6.6) in the presence or absence of Cu(II) for 24 h at 37 ℃. Oligomeric Aβ_{1-40} (Aβo) was used as a positive control.
