# Supplementary material for: Cu(II) enhances the effect of Alzheimer’s amyloid-β peptide on microglial activation
Source: J Neuroinflammation. 2015 Jun 24;12:122. doi: 10.1186/s12974-015-0343-3 (PMC4490619; doi:10.1186/s12974-015-0343-3)
Supplement: Additional file 2: Figure S2. — Characterization of the microglial culture purity. Microglial cultures were immunostained with the microglial marker CD11b. Scale bar = 300 μm. [file 12974_2015_343_MOESM2_ESM.pdf]

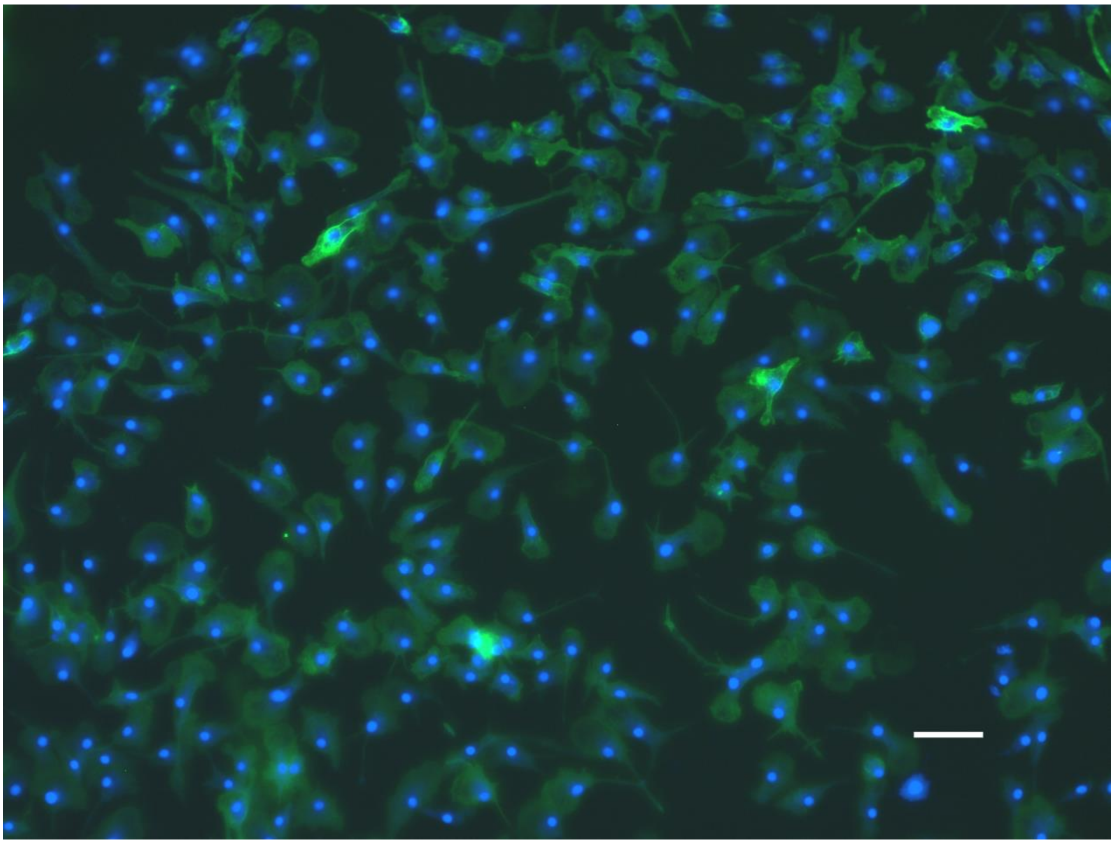

Additional file 2: Figure S2

Characterization of the microglial culture purity. Microglial cultures were immunostained with the microglial marker CD11b. Scale bar = 300 μm.
