# Supplementary material for: Cu(II) enhances the effect of Alzheimer’s amyloid-β peptide on microglial activation
Source: J Neuroinflammation. 2015 Jun 24;12:122. doi: 10.1186/s12974-015-0343-3 (PMC4490619; doi:10.1186/s12974-015-0343-3)
Supplement: Additional file 3: Figure S3. — Western blot analysis of iNOS expression in BV-2 cells. (A) Representative Western blots of iNOS in BV-2 cells after Cu(II)-Aβ1–40 (at 5 μΜ peptide concentration), Cu(II), or Aβ1–40 stimulation (for 24 h). (B) Densitometric analysis of iNOS/β-actin ratio normalized to saline control. n = 3 experiments. **P < 0.01 vs con; # P < 0.05 vs Cu-Aβ. [file 12974_2015_343_MOESM3_ESM.pdf]

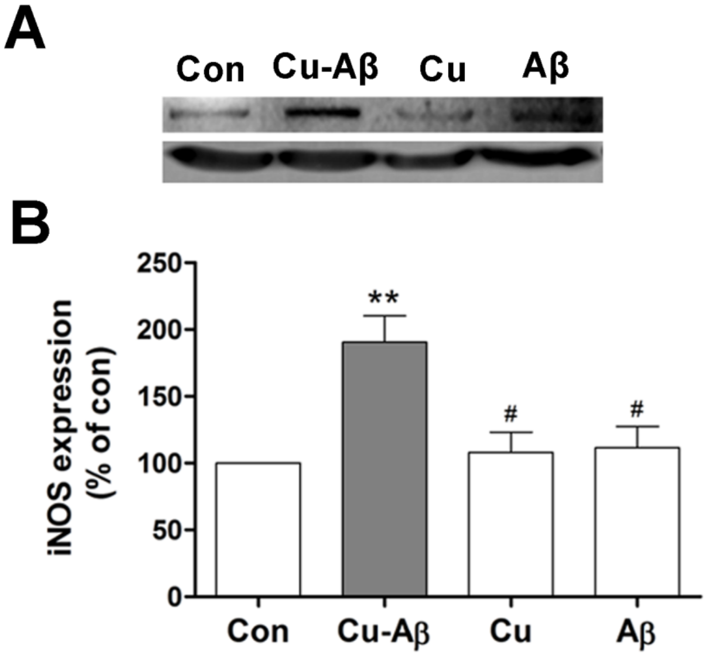

Additional file 3: Figure S3

Western blot analysis of iNOS expression in BV-2 cells. (A) Representative Western blots of iNOS in BV-2 cells after Cu(II)-Aβ_{1-40} (at 5 μM peptide), Cu(II), or Aβ_{1-40} stimulation (for 24 h). (B) Densitometric analysis of iNOS/β-actin ratio normalized to saline control. n = 3 experiments. $**P < 0.01$ vs con; $^{\#}P < 0.05$ vs Cu-Aβ.
